# Multidisciplinary management of chronic refractory pain in autosomal dominant polycystic kidney disease

**DOI:** 10.1093/ndt/gfac158

**Published:** 2022-05-25

**Authors:** Franka van Luijk, Ron T Gansevoort, Hans Blokzijl, Gerbrand J Groen, Robbert J de Haas, Anna M Leliveld, Esther Meijer, Joke M Perdok, Ruud Stellema, Andreas P Wolff, Niek F Casteleijn

**Affiliations:** Department of Nephrology, University Medical Center Groningen, University of Groningen, Groningen, The Netherlands; Department of Nephrology, University Medical Center Groningen, University of Groningen, Groningen, The Netherlands; Department of Gastroenterology and Hepatology, University Medical Center Groningen, University of Groningen, Groningen, The Netherlands; Department of Anesthesiology (Pain Center), University Medical Center Groningen, University of Groningen, Groningen, The Netherlands; Department of Radiology, University Medical Center Groningen, University of Groningen, Groningen, The Netherlands; Department of Urology, University Medical Center Groningen, University of Groningen, Groningen, The Netherlands; Department of Nephrology, University Medical Center Groningen, University of Groningen, Groningen, The Netherlands; Department of Anesthesiology (Pain Center), University Medical Center Groningen, University of Groningen, Groningen, The Netherlands; Department of Anesthesiology (Pain Center), University Medical Center Groningen, University of Groningen, Groningen, The Netherlands; Department of Anesthesiology (Pain Center), University Medical Center Groningen, University of Groningen, Groningen, The Netherlands; Department of Urology, University Medical Center Groningen, University of Groningen, Groningen, The Netherlands

**Keywords:** ADPKD, nephrectomy, nerve block, polycystic kidney disease

## Abstract

**Background:**

Chronic pain is often difficult to manage in autosomal dominant polycystic kidney disease (ADPKD) patients and sometimes even leads to nephrectomy. We analyzed the long-term efficacy of our innovative multidisciplinary protocol to treat chronic refractory pain that aims to preserve kidney function by applying among other sequential nerve blocks.

**Methods:**

Patients were eligible if pain was present ≥3 months with a score of ≥50 on a visual analog scale (VAS) of 100, was negatively affecting quality of life and if there had been insufficient response to previous therapies, including opioid treatment. Treatment options were, in order, analgesics, cyst aspiration and fenestration, nerve blocks and nephrectomy.

**Results:**

A total of 101 patients were assessed in our clinic (mean age 50 ± 11 years, 65.3% females). Eight patients were treated with medication, 6 by cyst aspiration or fenestration, 63 by nerve blocks and 6 received surgery as the first treatment option. Overall, 76.9% experienced a positive effect on pain complaints shortly after treatment. The VAS score was reduced from 60/100 to 20/100 (P < 0.001) and patients decreased their number of nonopioid and opioid analgesics significantly (P < 0.001, P = 0.01, respectively). A substantial number of the patients (*n* = 51) needed additional treatment. At the end of follow-up in only 13 patients (12.9%) was surgical intervention necessary: 11 nephrectomies (of which 10 were in patients already on kidney function replacement treatment), 1 liver transplantation and 1 partial hepatectomy. After a median follow-up of 4.5 years (interquartile range 2.5–5.3), 69.0% of the patients still had fewer pain complaints.

**Conclusions:**

These data indicate that our multidisciplinary treatment protocol appears effective in reducing pain in the majority of patients with chronic refractory pain, while postponing or even avoiding in most patients surgical interventions such as nephrectomy in most patients.

## Graphical Abstract

**Graphical Abstract:**
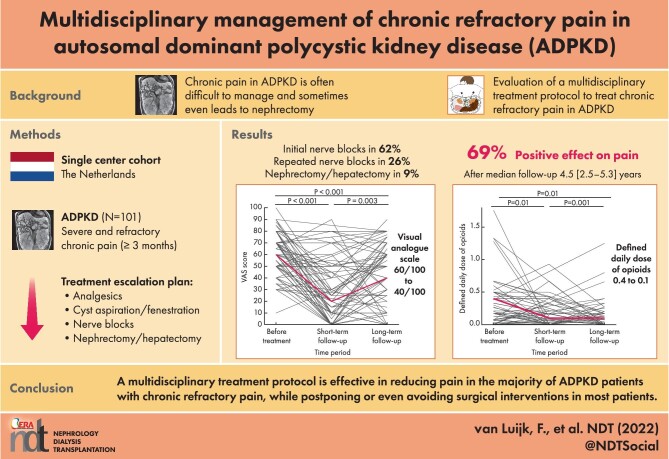

KEY LEARNING POINTS **What is already known about this subject?**

- Chronic pain is often difficult to manage in autosomal dominant polycystic kidney disease (ADPKD) patients and even leads to nephrectomy, which is problematic, because ADPKD is a progressive disease that leads to kidney failure in most patients.

**What this study adds?**

- In our expertise center for polycystic kidney disease, 101 patients with refractory pain complaints were treated, of which the majority (76.9%) experienced a positive effect on pain complaints shortly after treatment and after a median follow-up of 4.5 years; still, 69.0% of the patients had fewer pain complaints.

**What impact this may have on practice or policy?**

- Our multidisciplinary treatment protocol, by applying among other sequential nerve blocks, appears effective in reducing pain in the majority of patients with chronic refractory pain, while postponing or even avoiding in most patients surgical interventions such as nephrectomy and (partial) hepatectomy.

## INTRODUCTION

Pain is common in patients with autosomal dominant polycystic kidney disease (ADPKD) [1–3]. Around 60% of patients experience pain that is often reported to occur early in the disease course and results in diminished quality of life [1, 2, 4–6]. Pain can be acute or chronic, in case it exists for >3 months. Chronic pain can be difficult to manage and leads to a need for nephrectomy or liver transplantation in up to 30% of patients [7, 8]. This is problematic because ADPKD is a progressive disease that leads to kidney failure in most affected patients [9]. A nephrectomy before the onset of kidney failure will therefore lead to shortening of the time until the start of kidney replacement treatment (KRT).

**FIGURE 1:**
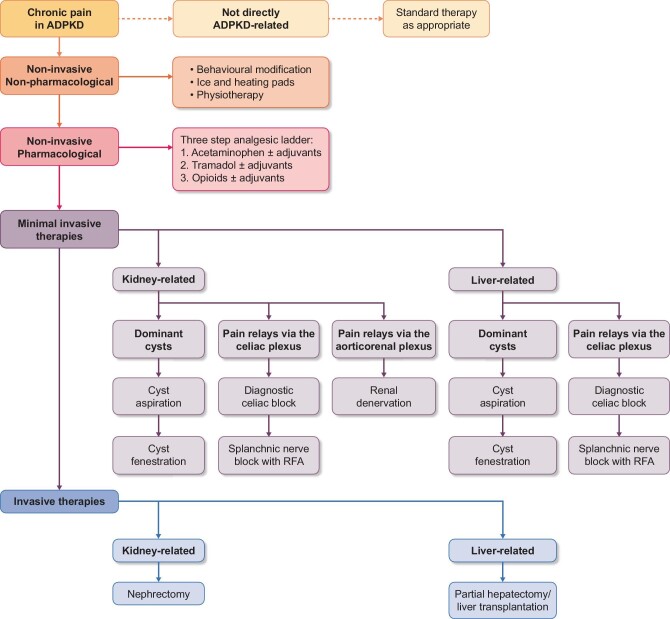
Treatment protocol for chronic refractory pain in ADPKD patients.

Since cysts are formed not only in the kidneys, but also in the liver in the majority of the patients, and pain can originate from diseases other than ADPKD, careful multidisciplinary assessment is needed to identify the cause and mechanism of pain and to offer the best treatment [3, 10]. In our polycystic kidney disease (PKD) expertise center, a novel multidisciplinary treatment protocol was introduced in 2013 in which we aim to spare kidney function by avoiding or postponing nephrectomy by applying sequential nerve blocks (Fig. 1) [8, 11].

This protocol is based on neuro anatomic knowledge that pain stimuli travel by different pathways to the central nervous system. In case of pain caused by pressure of the enlarged cystic organs on adjacent tissues or by distension of the hepatic capsule, pain stimuli are relayed via the celiac plexus and the major splanchnic nerves, whereas pain caused by distension of the renal capsule, the predominant relaying pathway, is via the aorticorenal plexus [12, 13]. By applying a diagnostic, temporary celiac nerve block with the local anesthetic lidocaine, it is possible to distinguish between pain that is related to distension of the hepatic capsule or compression of adjacent tissue versus pain related to distension of the renal capsule. When such a diagnostic nerve block is successful but pain recurs, long-term splanchnic nerve blocks are performed with radiofrequency ablation (RFA) [8, 14, 15].

Our initial results with this protocol were promising. In 2017 we reported that after a median follow-up of 12 months, 81.8% of the 44 patients who received nerve blocks experienced a sustained improvement in pain intensity, indicating that our treatment protocol was effective in providing pain relief in ADPKD patients with chronic refractory pain [11]. However, not all patients were eligible to undergo a nerve block. In these patients, other treatment options were considered and evaluated. In addition, the effect of nerve blocks may be temporary, because nerves can recover or pain stimuli can reroute after RFA [16]. In the present report we describe in >100 ADPKD patients with chronic, severe pain not only the short-term, but also the longer-term results of the applied nerve blocks as well as the results of the other treatment modalities.

## MATERIALS AND METHODS

### Study population

From August 2013 to August 2020, patients with ADPKD and chronic, severe pain were screened at the pain clinic of our expertise center for PKDs at the University Medical Center Groningen, The Netherlands. Some patients referred themselves, while others were referred by their treating physician from all over The Netherlands. All screened ADPKD patients were included in this analysis. Indications for referral were pain complaints with a pain score on a visual analog scale (VAS; 0–100) ≥50, that were likely to be ADPKD related, lasting ≥3 months, incapacitating (negatively affecting physical and/or social life according to self-assessment) and with insufficient response to previous pain therapies. The institutional review board concluded that this protocol was exempt from approval because it was considered to be a protocolized introduction of novel clinical care (METc 2013.299).

### Study assessment

After referral, intake took place by a nephrologist or urologist at the pain clinic of our PKD expertise center as well as by a pain specialist. All patients received a questionnaire before intake, including questions considering medication use, VAS score (scale 0–100) and two validated quality-of-life questionnaires [the nine-item patient health questionnaire (PHQ-9), which maps depressive symptoms, and the 36-item Short Form Health Survey (SF-36), which assesses quality of life]. The SF-36 can be divided into two domains: the physical component score (PCS) and the mental component score (MCS). These domains were scored from 0 to 100, with a higher score corresponding with a better quality of life. During intake, pain characteristics, nephrourological symptoms, medication use and medical history were discussed. If patients reported pain in one of their loins, lower back or abdomen, the pain was regarded to be of renal origin. If patients reported pain on the right side in the upper abdomen and behind or immediately below the ribcage, it was regarded to be of hepatic origin. Blood pressure was measured in a supine position with an automated oscillometric device (Dinamap, GE Healthcare Systems, Chicago, IL, USA). Blood and urine samples were collected. Per the protocol, magnetic resonance imaging (MRI) without contrast was performed and assessed by a radiologist experienced in PKD to exclude other causes of pain and to determine liver and kidney volume. In addition, a radioisotope renography was performed to exclude postrenal obstruction and afunctional kidneys. After this intake, patients were discussed with a multidisciplinary team to determine the most appropriate treatment according to the treatment protocol (Fig. 1). The multidisciplinary team included a nephrologist, urologist, pain specialist, radiologist and hepatologist. For special cases, a gynecologist or transplant surgeon were consulted.

### Treatment protocol

First, non-ADPKD-related pain was excluded by history, laboratory investigations, MRI and, if deemed necessary, additional investigations. Nonpharmacological therapies and analgesics were prescribed or optimized as a first step. In case of pain probably related to a polycystic liver and substantial enlargement of the liver, somatostatin analogs, such as lanreotide, were prescribed to reduce the rate of liver growth and pain complaints. These drugs inhibit cyclic adenosine monophosphate generation, leading to less cell proliferation and a decrease in liver volume [17].

If these therapies were unsuccessful and one or a few dominant cysts were observed, a cyst aspiration without sclerosant was performed. In case this was successful, this was followed by (robot-assisted) laparoscopic fenestration when pain recurred. In case the patient was KRT dependent and fit for surgery, native nephrectomy was the option of first choice. Treatment effect was assessed by the nephrologist or urologist 4 weeks after intervention and a VAS score was documented. If no dominant cysts were observed, the patient was not KRT dependent and treatment with nerve blocks was indicated, a diagnostic, temporary celiac plexus block was performed at the most painful side under local anesthetic. In short, the patient was placed in a prone position. A 20-gauge 15-cm spinal type needle (Cosman) was advanced from posterior to anterior toward the ventral surface of the L1 vertebral body under fluoroscopic guidance. After the needle position was confirmed by injection of contrast medium, 10 mL of bupivacaine (0.5%) was injected.

If the VAS score decreases to ≤30, the procedure is regarded as successful and patients are scheduled for a long-term splanchnic block with RFA when pain recurs. A 20-gauge, 15-cm spinal-type needle (Cosman RF) is advanced from posterior to anterior toward the ventral 1/3 surface of the vertebral body of Th11. Positioning takes place under fluoroscopic guidance and is deemed correct when there is bone contact. After the correct needle position is confirmed by injection of contrast medium, three applications of radiofrequency energy at 80^o^ C are executed. The time needed to perform a diagnostic celiac plexus block or RFA of the major splanchnic nerves 20–30 min. For both procedures patients are observed closely for 2–4 h postprocedure, including vital signs monitoring, until discharge. If pain relief is observed after the splanchnic RFA block but recurred after a significant period of time, a splanchnic block is repeated. If no substantial pain relief is seen after the diagnostic celiac plexus or splanchnic nerve block, patients underwent catheter-based renal denervation [11, 14, 15]. Of note, renal denervation was not available for patients from 2016 to 2021 because ablation catheters were no longer available due to the disappointing results of the Simplicity trials [18]. Two weeks after the diagnostic celiac plexus nerve block and 6 weeks after the long-term splanchnic block or renal denervation, patients were asked to fill out a questionnaire including questions about VAS score, medication use and quality of life (SF-36 and PHQ-9). Significant short-term pain reduction was defined as reaching a VAS score ≤30/100. The study protocol is discussed in more detail elsewhere [11].

In some patients, the above-mentioned treatment options were ineffective to achieve substantial or sustained pain relief and surgical interventions had to be considered. If the splanchnic RFA block was initially successful but pain recurred, a splanchnic RFA block could be repeated. In case of continuing pain and substantial enlargement of the liver and gastrointestinal as well as pain-related problems, a partial hepatectomy or a liver transplantation was considered. In case of continuing pain probably related to the polycystic kidneys, a nephrectomy was considered. Nephrectomy or hepatectomy was only performed for pain relief, not for other indications. Either the nephrologist, urologist or hepatologist assessed the efficacy after the intervention and a VAS score was documented. The multidisciplinary treatment protocol is depicted in Fig. 1.

### Long-term follow-up

All patients were asked to fill out a questionnaire in August 2020 to assess the long-term effect of the interventions performed in the framework of this treatment protocol. The follow-up questionnaire included a VAS score, a quality-of-life score (SF-36 and PHQ-9) and questions about additional pain treatments and medication use.

### Statistical analysis

Statistical analyses were performed using SPSS version 23.0 (IBM, Armonk, NY, USA). A two-sided P-value < 0.05 was considered significant. Normally distributed data are presented as mean ± standard deviation (SD), nonnormally distributed data as median [interquartile range (IQR)] and categorical data as percentages. A baseline characteristics table was created in which patients were grouped based on the type of treatment they received (Table 1). The different treatment types were (in respective order): (1) no treatment; (2) medication, physiotherapy and transcutaneous electrical nerve stimulation (TENS), analgesics or lanreotide; (3) cyst aspiration or cyst fenestration; (4) nerve blocks and (5) surgical intervention (nephrectomy, partial hepatectomy and liver transplantation). If patients received two types of treatment, they were included in both groups. For statistical analysis between the groups, patients who received more than one type of treatment were placed into the highest category, with medication being the lowest and surgical intervention being the highest category. P-values for differences between groups were tested with One-way analysis of variance (ANOVA) for normally distributed data, a Kruskal–Wallis test for nonnormally distributed data and Pearson chi-squared test or Fisher's test for categorical data.

**Table 1.** Baseline patient characteristics per treatment group

		Different treatment groups					
	Total	No treatment	Medication, physiotherapy	Cyst aspiration, fenestration	Nerve blocks	Nephrectomy (hemi)hepatectomy	P-value
Characteristics	(*N* = 101)	(*n* = 17)	(*n* = 21)	(*n* = 13)	(*n* = 64)	(*n* = 15)	
Female (%)	65.3	72.2	52.4	53.8	73.4	60.0	0.03
Age at baseline (years), mean ± SD	50 ± 11	54 ± 10	49 ± 13	48 ± 10	50 ± 10	52 ± 10	0.1
Body mass index, mean ± SD	27 ± 4	25 ± 5	27 ± 4	29 ± 4	26 ± 4	26 ± 5	0.2
History of (%)							
Urinary tract infection	60.8	82.4	42.9	41.7	65.1	50.0	0.01
Renal cyst infection	28.6	46.7	25.0	40.0	26.3	18.2	0.3
Liver cyst infection	7.9	14.3	–	9.1	5.0	23.1	0.1
Renal stones	9.2	–	11.1	9.1	11.9	16.7	0.5
Liver cysts	88.8	100.0	94.4	91.7	85.0	100.0	0.1
Systolic blood pressure (mmHg), mean ± SD	136 ± 15	136 ± 22	137 ± 15	137 ± 16	136 ± 15	135 ± 14	1.0
Diastolic blood pressure, mean ± SD	84 ± 10	83 ± 11	84 ± 12	83 ± 9	85 ± 9	81 ± 11	0.4
Use of blood pressure-lowering drugs (%)	74.0	61.1	76.2	84.6	74.6	80.0	0.7
Non-KFRT dependent (%)	76.5	47.1	81.0	100.0	88.9	73.3	<0.001
eGFR (mL/min/1.73 m^2^), mean ± SD	57 ± 23	48 ± 26	66 ± 21	66 ± 22	57 ± 20	50 ± 20	0.04
KFRT dependent (%)	23.5	52.9	19.0	–	11.1	26.7	0.01
Hemodialysis	30.4	55.6	0	–	–	50	0.01
Kidney transplantation	69.6	44.4	100	–	100	50	0.4
Organ volumes (mL), median (IQR)							
Left kidney	968 (578–1589)	856 (334–1564)	988 (665–2263)	1038 (748–1733)	898 (530–1416)	1179 (533–2193)	0.2
Right kidney	970 (556–1631)	934 (467–1965)	1210 (681–2188)	1229 (526–1741)	809 (517–1487)	1563 (434–2068)	0.1
Total kidney	1996 (1010–3193)	1765 (676–3275)	2222 (1316–4541)	2222 (1274–3777)	1715 (943–2745)	3072 (991–3969)	0.08
Liver	2464 (1964–3300)	2897 (2345–3594)	2344 (1780–3133)	2541 (2318–3335)	2481 (1754–3222)	2529 (2028–4285)	0.05
Total kidney and liver	4590 (3777–6169)	4590 (4083–5976)	4755 (3771–7805)	4663 (4488–6095)	4488 (3093–5824)	6036 (4533–7217)	0.03

All patients were divided into treatment groups. If patients received more than one type of treatment they were added to both groups. For analysis, patients were put into the highest category, with medication being the lowest and nephrectomy/(hemi)hepatectomy being the highest category. P-values for differences between groups were tested with one-way ANOVA for normally distributed data, Kruskal–Wallis test for nonnormally distributed data and Pearson chi-squared test or Fisher's test for categorical data. eGFR, estimated glomerular filtration rate.

Differences between preintervention and short-term follow-up and preintervention and long-term follow-up were tested with paired *t*-tests for normally distributed data and Wilcoxon signed rank test for nonnormally distributed data. VAS score, PCS score, MCS score, blood pressure and defined daily dose (DDD) medication were compared.

## RESULTS

**FIGURE 2:**
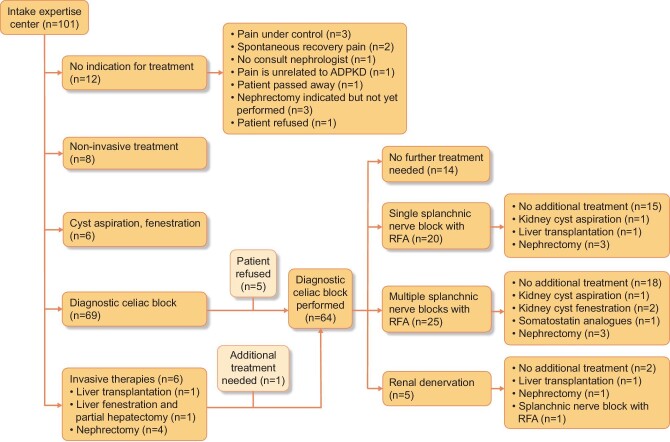
Flowchart of patients included for protocolized treatment of chronic refractory pain in ADPKD.

In total, 101 patients were assessed at our ADPKD pain clinic. Patients were referred from different parts of The Netherlands and sometimes from abroad (Supplementary data, Figure S1). Figure 2 provides a flowchart describing the various treatment options that were offered. Seventeen patients did not receive any treatment. Most of these patients (*n* = 14) experienced spontaneous pain recovery or refused treatment. In one patient, pain was not directly related to ADPKD. Of the remaining patients, a total of 64 patients were treated with nerve blocks, 8 patients were treated with medication and physiotherapy, 6 patients received cyst aspiration and/or fenestration (5 renal cysts and 1 liver cyst) and 6 patients received surgical interventions as the first therapy (among which 4 were nephrectomies, all in patients who were KRT dependent) (Table 1). In six patients, somatostatin analogues were prescribed to reduce volume-related pain complaints, especially liver-related symptoms.

### Patient and pain characteristics

Overall, the mean age of patients referred to our pain clinic was 50 ± 11 years and 65.3% were females (Table 1). A total of 76.5% of patients were non-KRT dependent, with a mean glomerular filtration rate of 57 ± 23 mL/min/1.73 m^2^. Five patients used tolvaptan, a vasopressin 2 receptor antagonist, to ameliorate kidney volume growth and to preserve renal function decline [4]. The remaining 23.5% of patients were KFRT dependent (30.4% on hemodialysis and 69.6% living with a kidney transplant). The median duration of pain was 5 years (IQR 2–12) and the overall median VAS score was 60 (IQR 50–80). Nonpharmacological therapies, predominantly physiotherapy, had been tried in 57.5% of the patients, 41.9% used mild opioids, 43.0% used strong opioids and 32.2% had already undergone pain therapies, such as cyst aspiration (10.9%), cyst fenestration (4%) or contralateral nephrectomy (2%). Comparing the five treatment subgroups showed that there was a significantly larger total kidney and liver volume in patients who underwent surgical interventions compared with the other groups (P = 0.03) (Table 1). The duration of pain was significantly longer in both the cyst aspiration group and in the patients who received nerve blocks, with a median pain duration of 6 years (IQR 2–16) and 6 years (IQR 3–18) (both P = 0.04 when compared with medication/physiotherapy group), respectively. No difference was observed in VAS scores and reported pain locations between the different groups (Table 2).

**Table 2.** Baseline pain characteristics per treatment group

		Treatment groups					
	Total	No treatment	Medication, physiotherapy	Cyst aspiration, fenestration	Nerve blocks	Nephrectomy(hemi)hepatectomy	P-value
Characteristics	(*N* = 101)	(*n* = 17)	(*n* = 21)	(*n* = 13)	(*n* = 64)	(*n* = 15)	
Duration of pain (years), median (IQR)	5 (2–12)	4 (2–11)	3 (1–9)	6 (2–16)	6 (3–18)	2 (1–6)	0.1
Duration of refractory pain (months), median (IQR)	12 (12–24)	24 (6–29)	12 (4–12)	12 (12–30)	12 (12–24)	12 (12–39)	0.4
Pain severity last 4 weeks, median (IQR)							
Minimum VAS score (0–100)	40 (20–60)	20 (0–40)	30 (25–58)	40 (20–60)	40 (25–60)	40 (23–50)	0.2
Maximum VAS score (0–100)	80 (70–90)	75 (55–94)	80 (60–91)	80 (70–90)	85 (80–90)	85 (71–90)	0.4
Average VAS score (0–100)	60 (50–80)	50 (33–78)	60 (50–70)	60 (53–70)	65 (50–80)	55 (48–70)	0.3
Patient reported location (%)							
Left kidney	23.8	16.7	9.5	23.1	28.1	26.7	0.4
Right kidney	20.8	27.8	23.8	23.1	18.8	6.7	0.5
Bilateral	46.5	38.9	61.9	46.2	45.3	53.3	0.9
Liver	30.7	38.9	47.1	30.8	34.4	41.7	0.6
SF-36 score, mean ± SD							
Physical component score (0–100)	58 ± 23	54 ± 25	55 ± 22	60 ± 26	58 ± 21	53 ± 26	0.5
Mental component score (0–100)	65 ± 20	58 ± 16	68 ± 25	67 ± 20	63 ± 20	63 ± 20	0.3
PHQ-9 total score, median (IQR)	9 (6–15)	11 (7–15)	8 (4–15)	12 (8–18)	9 (6–16)	9 (8–13)	0.4
Management of pain (%)							
Nonpharmacological therapies	57.5	28.6	41.2	45.5	70.0	75.0	0.02
Acetaminophen	77.9	78.6	75.0	80.0	78.0	85.7	0.7
NSAIDs	5.8	7.1	12.5	–	5.1	–	0.5
Mild opioids	41.9	35.7	43.8	30.0	40.7	85.7	0.01
Strong opioids	43.0	50.0	31.3	50.0	44.1	14.3	0.1
Previous pain therapies	32.2	28.6	17.6	45.5	40.0	16.7	0.1
Nerve bock	2.0	–	4.8	–	3.1	–	0.9
Cyst aspiration	13.9	17.6	4.8	7.7	17.2	–	0.2
Cyst sclerotherapy or fenestration	7.9	5.9	9.5	15.4	7.0	–	0.5
Nephrectomy or (hemi) hepatectomy	2.0	5.9	–	–	1.6	–	0.9

All patients were divided into treatment groups. If patients received more than one type of treatment, they were added to both groups. For analysis, patients were put into the highest category, with medication being the lowest and nephrectomy/(hemi)hepatectomy being the highest category. P-values for differences between groups were tested with one-way ANOVA for normally distributed data, Kruskal–Wallis test for nonnormally distributed data, and Pearson chi-squared test or Fisher's test for categorical data.

### Protocolized treatment

In 76.9% of all treated patients a significant short-term pain reduction (defined as reaching a VAS score ≤30/100) could be achieved by the initial treatment option (Table 3). The median change in VAS score pre- and 2–6 weeks post intervention was 40/100 (IQR 8–60; P < 0.001), which corresponds to a decrease of 71% (IQR 13–100). The use of analgesics was significantly reduced after the interventions, with a decrease in the median dose of nonopioids and opioids (1.2 ± 0.4 versus 0.8 ± 0.7 DDD, P = 0.001 and 0.4 ± 0.4 versus 0.1 ± 0.2 DDD, P = 0.01, respectively). Overall, no difference between preintervention and short-term post intervention quality of life was measured [change in PCS +5 (IQR 0–10), P = 0.5; change in MCS −4 (IQR −6–10), P = 0.3; change in PHQ-9 +0 (IQR −1–2), P = 0.5] (Table 3). In each of the treatment groups, a significantly lower VAS score was observed after the intervention (Supplementary data, Table S1). Of the patients who were treated with nerve blocks, 74.2% experienced a positive effect by the intervention. The median decrease in VAS score pre- and shortl-term postintervention was 40/100 (IQR 3–60; P < 0.001). The doses of opioids and non-opioids were significantly lower postintervention compared with preintervention (1.2 ± 0.5 versus 0.8 ± 0.7 DDD, P = 0.001 and 0.4 ± 0.5 versus 0.1 ± 0.2 DDD, P = 0.006, respectively). Of the patients who received medication or physiotherapy, 94.1% reported a positive effect for their last intervention. The median change in VAS score pre- and postintervention was 40/100 (IQR 28–58; P = 0.001). Patients who received a cyst aspiration or fenestration noted a positive effect for their last treatment in 84.6% of the cases. The difference in VAS score pre- and postintervention was 60/100 (IQR 15–60; P = 0.01). Lastly, 93.3% of patients who received surgical intervention reported a positive effect for their last intervention, with a median change in VAS score of 50 (IQR 30–60; P = 0.001) (Supplementary data, Table S2).

**Table 3.** Overall results of last pain treatment, short-term follow-up and long-term follow-up

Overall (*N* = 83)	Before intervention	Short-term follow-up	Long-term follow-up	P-value before versus short	P-value before versus long
Positive effect last intervention (%)	–	76.9	69.0	–	–
VAS score (0–100), median (IQR)	60 (50–80)	20 (0–50)	40 (10–60)	<0.001	<0.001
Defined daily dose nonopioids, mean ± SD	1.2 ± 0.4	0.8 ± 0.7	0.4 ± 0.7	0.001	0.001
Defined daily dose opioids, mean ± SD	0.4 ± 0.4	0.1 ± 0.2	0.1 ± 0.2	0.01	0.01
Physical component score (0–100), mean ± SD	59 ± 22	61 ± 23	63 ± 24	0.5	0.4
Mental component score (0–100), mean ± SD	67 ± 20	64 ± 21	71 ± 17	0.3	0.03
PHQ-9 score (0–27), median (IQR)	9 (6–15)	8 (4–13)	6 (3–11)	0.5	0.001

### Need for additional treatment and scaling up with surgical interventions as a final option

A substantial part of the overall group of 101 patients needed additional treatment after the initial intervention (*n* = 51). In case a diagnostic celiac block led to significant pain reduction but pain recurred (*n* = 45), patients underwent a long-term splanchnic nerve block with RFA. Of these 45 patients, 25 (55.6%) needed multiple splanchnic nerve block. There were two reasons to perform repeat splanchnic nerve blocks. First was because the initial RFA block did not achieve sufficient pain relief and another RFA block had to be performed (*n* = 6). In five of these patients the next RFA block was successful. Second, it could be that pain recurred after an initial successful RFA block (*n* = 19). In this latter group the median number of splanchnic nerve blocks performed per patient was 3 (IQR 2–4), with 1 patient receiving seven splanchnic nerve blocks, which all had a good effect, but temporary. If pain recurred after an initial successful RFA block, the median time between the intervention and recurrence of pain complaints was 6 months (IQR 3–12). In such cases, pain was initially mild but steadily increased, leading to a repeat block 9 months (IQR 6–13) after the previous block.

Remarkably, and an a priori unexpected observation, in 14 patients, chronic pain complaints did not recur after the initial temporary diagnostic celiac block, i.e. pain remained below a VAS of 30/100 and no further action had to be taken. Catheter-based renal denervation was indicated in nine patients, however, only five patients underwent this procedure because the renal denervation catheters were not available between 2016 and 2021. In these five patients, renal denervation led to substantial pain relief in three of the patients and an overall decrease in median VAS score from 60/100 to 20/100 (P = 0.07).

**FIGURE 3:**
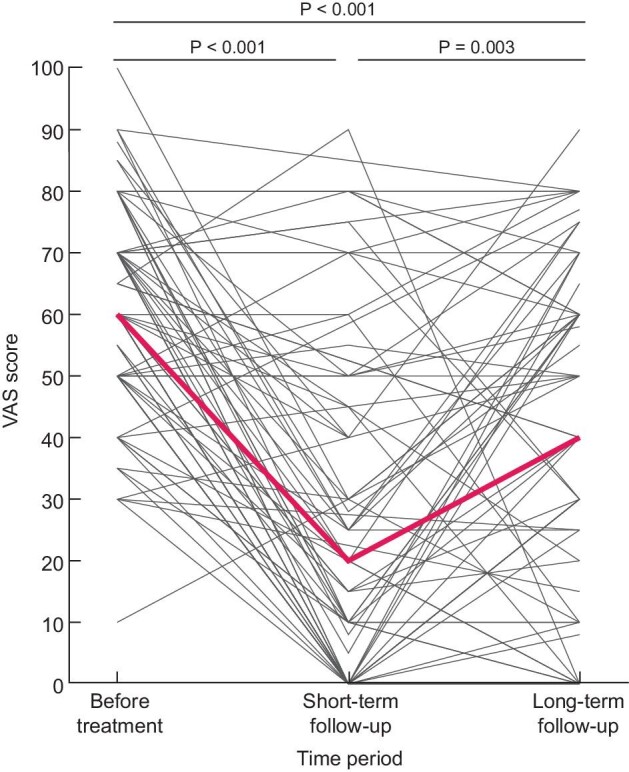
Effect of pain treatment on VAS score shortly after the intervention (2–6 weeks) and after longer-term follow-up. The red line indicates the median VAS score.

In a relatively small number of patients [*n* = 14 (13.8%)] nerve blocks did not achieve sufficient pain relief and there was a need for scaling up (Fig. 3). Four patients underwent a subsequent cyst aspiration that was successful in three patients (decrease in median VAS score 60/100 to 20/100). In two patients, pain recurred and a second procedure with cyst fenestration was performed with a significant beneficial effect on pain in both patients (decrease in median VAS score from 50/100 to 10/100). Two patients underwent a hepatectomy with liver transplantation after sequential nerve block due to the recurrence of pain complaints after a nerve block. Nephrectomy was performed in seven patients (of whom four were already KFRT dependent and one patient had an afunctional native kidney on renography at the time of nephrectomy). In patients who underwent hepatectomy with liver transplantation or native nephrectomy the VAS score decreased significantly [VAS before treatment 55 (IQR 40–70) versus VAS shortly after intervention 0 (IQR 0–10), P = 0.001].

### Long-term follow-up

**FIGURE 4:**
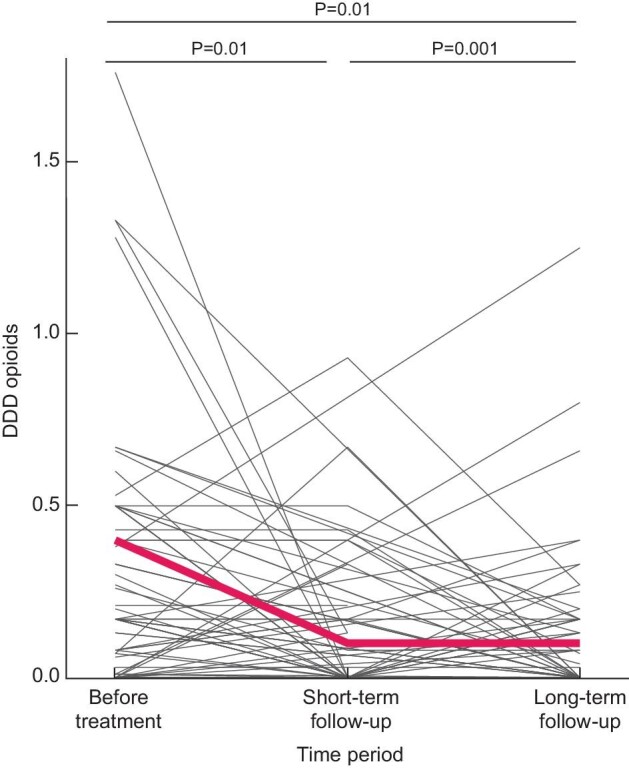
Effect of pain treatment on the use of opioids shortly after the intervention (2–6 weeks) and after longer-term follow-up. The red line indicates the mean DDD opioid use.

All patients were followed per protocol until August 2020, with a median follow-up of 4.5 years (IQR 2.5–5.3). Overall, VAS scores were still significantly decreased compared with the VAS scores preintervention (P < 0.001). However, patients reported a lower VAS score shortly after intervention compared with long-term follow-up (P = 0.003) (Fig. 3). Moreover, on long-term follow-up patients used less nonopioids and opioids compared with preintervention, but also when compared with the situation shortly after the intervention. Their analgesic use was significantly lower [DDD pre- versus short- versus long-term after the intervention for nonopioids (1.2 ± 0.4 versus 0.8 ± 0.7, P = 0.001 versus 0.4 ± 0.7, P = 0.001), and for opioids (0.4 ± 0.4 versus 0.1 ± 0.2, P = 0.01 versus 0.1 ± 0.2, P = 0.01] (Fig. 4). Daily opioid use was stopped in 28.1% of the patients and 36 patients (40.4%) reduced their opioid use. Neither patient characteristics or treatment choice were associated with a reduction or cessation of opioid use.

The PHQ-9 score at long-term follow-up was significantly lower compared with short-term follow-up, indicating that patients experienced fewer depressive symptoms [9 (IQR 6–15) versus 6 (IQR 3–11), P = 0.001]. In addition, an increase in mental health status was observed (MCS preintervention: 67 ± 20; MCS long-term: 71 ± 17; P = 0.03) and no differences were noticed in physical health (PCS preintervention: 59 ± 22; PCS long-term: 63 ± 24; P = 0.4). When analyzing the treatment subgroups separately, similar results were observed. However, in the nerve block subgroup, PCS at long-term follow-up was significantly better compared with preintervention (68 ± 23 versus 58 ± 21; P = 0.01).

### Adverse events

Some adverse events were observed after the diagnostic celiac blocks and splanchnic RFA blocks. A small number of patients (*n* = 4) experienced orthostatic hypotension or diarrhea after the first diagnostic celiac plexus block, but these adverse events were all self-limiting. In case blood was aspirated during the nerve block, the procedure was interrupted and repeated after 4 weeks (*n* = 2, both successful). A number of patients (*n* = 11) experienced the procedure, which was performed under local anesthesia, itself as painful. However, these procedures could all be finished successfully and procedure-related pain was only short-lasting and needed no intervention. In one of the five renal denervation procedures, the procedure was stopped because of a spasm of the renal artery. This intervention was repeated successfully 3 months later. No late complications occurred after the renal denervation procedures. Renal denervation resulted in a decrease in systolic blood pressure (134 ± 6 mmHg versus 126 ± 9; P = 0.01), but not in diastolic blood pressure (86 ± 6 mmHg versus 83 ± 9; P = 0.2). No complications were noted in patients who received cyst aspiration, cyst fenestration or nephrectomy.

## DISCUSSION

In the present study we describe the short- and long-term results of a multidisciplinary treatment protocol in 101 ADPKD patients with chronic refractory pain. The majority of patients treated according to this protocol experienced a positive effect on pain shortly after treatment. Even after a median follow-up of 4.5 years, 69.0% of the patients had fewer pain complaints and their analgesic use was significantly reduced.

In 2014 we introduced a new multidisciplinary treatment protocol in which a prominent place was set for sequential nerve blocks to avoid or postpone the need for nephrectomy in ADPKD [8]. Despite encouraging results, several limitations have to be mentioned. Nerve blocks were not performed in 26% of the patients, indicating that a substantial number of the patients needed other treatment options. In addition, long-term results on pain relief and safety were not available, including the need for repeat procedures or scaling up.

To address these limitations, we evaluated in the present study long-term follow-up of all patients with chronic refractory pain who were treated in our PKD expertise center. After a median follow-up time of 4.5 years, significant pain relief was observed, assessed as a decrease in VAS score as well as a decrease in the use of opioids and nonopioids. It should be noted that patients experienced a slightly higher VAS score at the long-term follow-up compared with shortly after the intervention. This minor increase in VAS score, however, had no negative effect on quality of life, and patients experienced even fewer depressive symptoms and better mental health status at the end of follow-up when compared with preintervention. This higher VAS score may have several reasons. First, chronic pain may have recurred after a new acute pain event, such as a cyst bleeding, infection or kidney stone. It is assumed that acute pain events can contribute to chronic pain due to central sensitization [13]. Also in our study group, pain recurrence was noticed in eight cases related to such an acute pain event. Second, it is known that nerve blocks can be temporary, because nerves can recover or pain stimuli can reroute after RFA [16]. Therefore some patients who underwent an RFA splanchnic block needed a repeat block (*n* = 19) that in most cases was successful (*n* = 16). Third, analgesic use was lower during long-term follow-up compared with short-term follow-up. Dealing with pain complaints is a delicate balance. It may be that the side effects of the analgesics outweighed the advantages of pain relief [6, 12, 19]. Coping with pain could mean that patients accept a higher VAS score because they are then less dependent on opioids and consequently experience fewer opioid- related side effects. Fourth, due to the progressive nature of the disease, kidney and liver volume increases steadily and can cause more compression of abdominal organs [8, 20]. Tolvaptan, a vasopressin 2 receptor antagonist, is now available to ameliorate kidney volume growth in ADPKD patients with rapid disease progression [4]. Data from the TEMPO 3:4 trial showed that tolvaptan use also reduced the number of acute pain events in ADPKD patients, but no data are available on the effect of tolvaptan on chronic pain complaints [21].

Some minor adverse events were reported after the various treatment procedures. However, no procedure-related severe adverse events were observed. As mentioned before, pain sensation can be impaired after treatment with nerve blocks and patients may therefore present with different symptomatology when suffering from abdominal diseases, which may delay making a correct diagnosis [11, 16, 22]. During follow-up, however, no delayed or missed diagnoses were reported, suggesting that the use of nerve blocks is relatively safe.

We observed that a substantial part of our study population is KFRT dependent (23.5%). It is sometimes assumed that chronic refractory pain in ADPKD is less common in KFRT-dependent patients, since kidney volume decreases after kidney transplantation or starting dialysis [23]. However, ADPKD-related pain is not associated with kidney or liver volume [20]. The high proportion of KFRT patients in our cohort may thus be related to the fact that patients are referred from all over The Netherlands to our expertise center. It should be noted though that most of these patients had long-standing pain that started well before the start of KFRT but was not treated satisfactory or resolved spontaneously. Furthermore, in our experience, chronic pain is an underestimated symptom in KFRT-dependent ADPKD patients that is not systematically assessed by many treating physicians and therefore often remains unnoticed.

Remarkably, in 14 patients the diagnostic celiac block resulted in substantial and sustained pain relief, in some patients even up to 7 years. This was unexpected, because lidocaine, the local anesthetic that is used for this procedure, is only able to block the sensory pathway temporarily. A possible explanation for this finding may be an effect on central sensitization caused by a short period of extreme nociceptive stimulation in the past, e.g. from a cyst infection or cyst bleeding. We assume that by applying local anesthetics, the continuous excitation of visceral nociceptive neurons is temporarily interrupted, allowing the neurons to return to their normal resting potential [24].

The aim of our protocol was to avoid or postpone nephrectomy in a disease that leads to kidney failure in most affected subjects [9]. A nephrectomy before the onset of kidney failure will considerably shorten the time until the start of KRT. Overall, only 11 patients of the 101 referred patients underwent nephrectomy, of which 7 were KRT dependent. In the remaining four patients, one patient had an afunctional kidney and three patients were already in the workup for a kidney transplantation. These data suggest that nerve blocks that are part of our protocol can avoid or postpone the need for nephrectomy in ADPKD. It must be noted, however, that our study did not contain a control group, thus our data may overestimate the positive effect of our treatment protocol on preserving kidney function in ADPKD patients with chronic refractory pain.

What may be the consequences of our findings? In our multidisciplinary protocol we performed individualized patient care. All patients were discussed by the multidisciplinary team and, based on the assessment by representatives of the various disciplines, the most kidney function-sparing treatment option was chosen with the a priori best chance of success. Our evaluation showed that this strategy is effective in reducing pain in the majority of patients with chronic refractory pain. Given the favorable efficacy:safety ratio, we propose that this protocol should be incorporated in clinical care. Because expertise and experience are needed by the various disciplines involved, and because the incidence of chronic, refractory pain is limited, we propose that implementation should be limited to PKD expertise centers. Furthermore, given the favorable efficacy:safety ratio, offering nerve blocks to ADPKD patients with less severe kidney-related pain should be considered, as well as to non-ADPKD patients. Other patients with chronic, refractory kidney pain related to a nonmalignant and noninfectious cause, such as loin pain hematuria syndrome or symptomatic parapelvic cysts, may also benefit [14, 25].

Of note, renal denervation was not available as a treatment option for patients from 2016 to 2021 because ablation catheters were no longer available due to the disappointing results of the Simplicity trials [20]. Ten patients were eligible for renal denervation, but due to the unavailability of devices, this procedure could only be performed in five patients, with substantial pain relief in three patients. In one patient, pain complaints recurred, with the result that three patients needed additional treatment. At the moment, limited data are available about the effect of catheter-based renal denervation on chronic pain in ADPKD patients [15]. Recently, new renal denervation devices have become available. Whether these will have a place in the management of pain remains to be studied.

This study has limitations, of which the most important is that it is a nonrandomized single-center experience. Short-term benefit akin to a placebo effect can therefore not be excluded. We chose to perform our protocol in such a setting because we considered it unethical to perform sham procedures in patients with chronic refractory pain, in line with the literature on placebo anesthetic blocks [26]. Second, some patients in this study were also included in our previous publication; however, in this previous study we only reported the short-term results of the first 44 patients. [11]. Third, per the protocol, quality of life was only assessed when nerve blocks were performed, but a VAS score was collected in all cases after an intervention. Fourth, in case of missing information after treatment, patients were approached retrospectively for data collection, which may have led to recall bias. The main strength of our study is the novelty of the approach, including various types of nerve blocks as kidney function–sparing treatment options, and the systematic and prospective nature of the data collection, including information on quality of life, resulting in a well-characterized population.

In conclusion, our multidisciplinary stepwise treatment protocol is effective in reducing pain in the majority of patients with chronic refractory pain and contributes to quality of life, while avoiding in nearly all patients surgical interventions such as nephrectomy of a functioning kidney and (partial) hepatectomy. We propose that our protocol should be implemented in the clinical care of PKD expertise centers and, given the satisfactory efficacy:safety ratio, also ADPKD patients with less severe pain may be eligible, and even non-ADPKD patients with similar complaints.

## Supplementary Material

gfac158_Supplemental_Files
