# Supplementary material for: Multidisciplinary management of chronic refractory pain in autosomal dominant polycystic kidney disease
Source: Nephrol Dial Transplant. 2022 May 25;38(3):618–29. doi: 10.1093/ndt/gfac158 (PMC9976741; doi:10.1093/ndt/gfac158)
Supplement: gfac158_Supplemental_Files [file gfac158_supplemental_files.zip › Supplementary Tables DEF.docx]

**Supplementary Table 1.**  Results per treatment modality, short-term follow-up

|  | **Before**  **Intervention** | **Short-term**  **Follow-up** | **P-value** |
| --- | --- | --- | --- |
|  |  |  |  |
| **Medication/Physiotherapy (n=21)** |  |  |  |
| Positive effect last intervention (%) | - | 94.1 | - |
| VAS score (0-100) | 60 [50-70] | 18 [0-48] | 0.001 |
| Defined Daily Dose non-opioids | 1.2 ± 0.4 | N/A | - |
| Defined Daily Dose opioids | 0.3 ± 0.1 | N/A | - |
| Physical Component Score (0-100) | 55 ± 22 | N/A | - |
| Mental Component Score (0-100) | 68 ± 25 | N/A | - |
| PHQ-9 score (0-27) | 8 [4-15] | N/A | - |
| **Cyst aspiration/fenestration (n=13)** | | |  |
| Positive effect last intervention (%) |  | 84.6 | - |
| VAS score (0-100) | 60 [55-70] | 23 [0-45] | 0.01 |
| Defined Daily Dose non-opioids | 1.2 ± 0.4 | N/A | - |
| Defined Daily Dose opioids | 0.3 ± 0.1 | N/A | - |
| Physical Component Score (0-100) | 62 ± 26 | N/A | - |
| Mental Component Score (0-100) | 65 ± 20 | N/A | - |
| PHQ-9 score (0-27) | 12 [8-18] | N/A | - |
| **Nerve blocks (n=64)** |  |  |  |
| Positive effect last intervention (%) |  | 74.2 | - |
| VAS score (0-100) | 65 [50-80] | 20 [7-50] | <0.001 |
| Defined Daily Dose non-opioids | 1.2 ± 0.5 | 0.8 ± 0.7 | 0.001 |
| Defined Daily Dose opioids | 0.4 ± 0.5 | 0.1 ± 0.2 | 0.006 |
| Physical Component Score (0-100) | 60 ± 21 | 61 ± 23 | 0.5 |
| Mental Component Score (0-100) | 63 ± 20 | 64 ± 21 | 0.3 |
| PHQ-9 score (0-27) | 9 [6-16] | 8 [4-12] | 0.5 |
| **Nephrectomy/(hemi)hepatectomy (n=15)** |  |  |  |
| Positive effect last intervention (%) |  | 93.3 | - |
| VAS score (0-100) | 55 [40-70] | 0 [0-10] | 0.001 |
| Defined Daily Dose non-opioids | 1.2 ± 0.5 | N/A | - |
| Defined Daily Dose opioids | 0.4 ± 0.5 | N/A | - |
| Physical Component Score (0-100) | 58 ± 21 | N/A | - |
| Mental Component Score (0-100) | 63 ± 20 | N/A | - |
| PHQ-9 score (0-27) | 9 [6-16] | N/A | - |
| Patients were divided into treatment groups. If patients received more than one type of treatment, they were added to both groups. Before intervention represents data collected during screening and short-term follow-up represents data collected 2-6 weeks after the last intervention. (*Abbreviations: n, number; VAS score, visual analogue scale score; PHQ-9, patient health questionnaire.)* | | | |

**Supplementary Table 2.** Overall results last pain treatment, long-term follow-up

|  | **Before**  **Intervention** | **Long-term**  **Follow-up** | **P-value** |
| --- | --- | --- | --- |
|  |  |  |  |
| **Medication/physiotherapy (n=21)** |  |  |  |
| VAS score (0-100) | 60 [50-70] | 50 [20-60] | 0.2 |
| Defined Daily Dose non-opioids | 1.2 ± 0.4 | 0.4 ± 0.6 | 0.005 |
| Defined Daily Dose opioids | 0.3 ± 0.1 | 0.1 ± 0.2 | 0.007 |
| Physical Component Score (0-100) | 55 ± 22 | 57 ± 23 | 0.5 |
| Mental Component Score (0-100) | 68 ± 25 | 70 ± 18 | 0.2 |
| PHQ-9 score (0-27) | 8 [4-15] | 7 [3-11] | 0.07 |
| **Cyst aspiration/fenestration (n=13)** | |  |  |
| VAS score (0-100) | 60 [55-70] | 50 [40-73] | 0.9 |
| Defined Daily Dose non-opioids | 1.2 ± 0.4 | 0.8 ± 0.5 | 0.4 |
| Defined Daily Dose opioids | 0.3 ± 0.1 | 0.4 ± 0.5 | 0.6 |
| Physical Component Score (0-100) | 62 ± 26 | 53 ± 18 | 0.6 |
| Mental Component Score (0-100) | 65 ± 20 | 69 ±21 | 0.9 |
| PHQ-9 score (0-27) | 12 [8-18] | 10 [5-11] | 0.3 |
| **Nerve blocks (n=64)** |  |  |  |
| VAS score (0-100) | 65 [50-80] | 40 [13-63] | <0.001 |
| Defined Daily Dose non-opioids | 1.2 ± 0.5 | 0.4 ± 0.7 | <0.001 |
| Defined Daily Dose opioids | 0.4 ± 0.5 | 0.1 ± 0.3 | 0.002 |
| Physical Component Score (0-100) | 60 ± 21 | 64 ± 23 | 0.2 |
| Mental Component Score (0-100) | 63 ± 20 | 70 ± 18 | 0.04 |
| PHQ-9 score (0-27) | 9 [6-16] | 7 [3-12] | 0.001 |
| **Nephrectomy/(hemi)hepatectomy (n=15)** |  |  |  |
| VAS score (0-100) | 55 [40-70] | 19 [0-60] | 0.01 |
| Defined Daily Dose non-opioids | 1.2 ± 0.5 | 0.1 ± 0.4 | 0.002 |
| Defined Daily Dose opioids | 0.4 ± 0.5 | 0.2 ± 0.4 | 0.1 |
| Physical Component Score (0-100) | 58 ± 21 | 57 ± 34 | 0.8 |
| Mental Component Score (0-100) | 63 ± 20 | 72 ± 19 | 0.5 |
| PHQ-9 score (0-27) | 9 [6-16] | 4 [1-11] | 0.04 |
| **No treatment performed (n=17)** |  |  |  |
| VAS score (0-100) | 55 [35-75] | 50 [40-77] | 0.9 |
| Defined Daily Dose non-opioids | 1.0 ± 0.5 | 0.5 ± 0.5 | 0.2 |
| Defined Daily Dose opioids | 0.4 ± 0.3 | 0.1 ± 0.1 | 0.3 |
| Physical Component Score (0-100) | 54 ± 25 | 64 ± 31 | 0.8 |
| Mental Component Score (0-100) | 58 ± 16 | 60 ± 22 | 0.9 |
| PHQ-9 score (0-27) | 11 [7-15] | 7 [2-8] | 0.7 |
| Patients were divided into treatment groups. If patients received more than one type of treatment, they were added to both groups. The control group represents the patient group that did not receive any treatment. *(Abbreviations: n, number; VAS score, visual analogue scale score; PHQ-9, patient health questionnaire.)* | | | |
