# Supplementary figures and images for: Multidisciplinary management of chronic refractory pain in autosomal dominant polycystic kidney disease
Source: Nephrol Dial Transplant. 2022 May 25;38(3):618–29. doi: 10.1093/ndt/gfac158 (PMC9976741; doi:10.1093/ndt/gfac158)

## Patient map

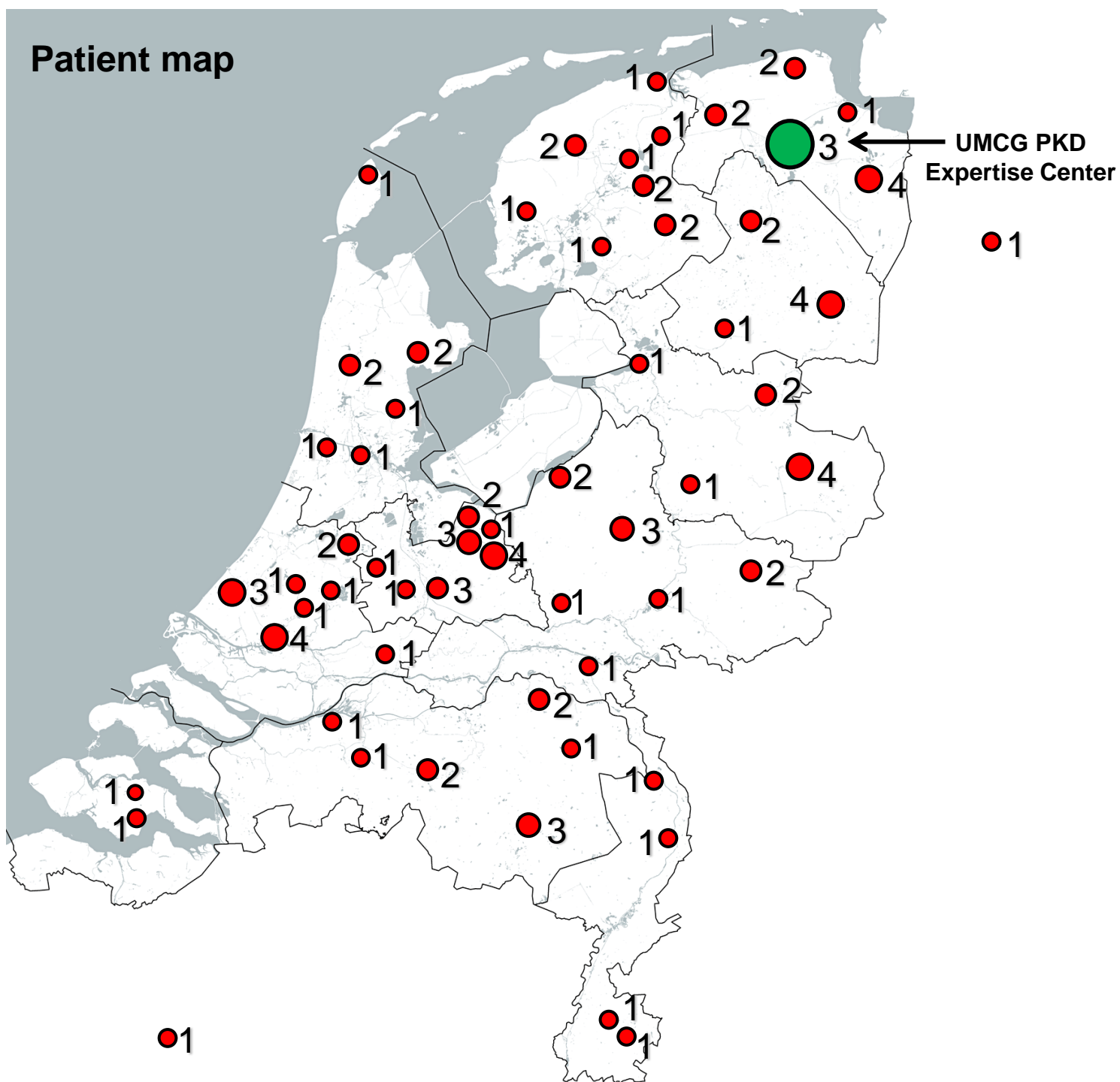

Supplement: gfac158_Supplemental_Files [file gfac158_supplemental_files.zip › Figure map DIPAK.pdf]
